# The Case Book, or Dentist's Day-Book, Leger, and Recorder, Combined

**Published:** 1847-10

**Authors:** 


*The Case-Book, or Dentist's Day-Book, Leger, and Recorder, combined.* Containing a system for Recording Operations on the Teeth, by which the surface and even the location of the surface may be recorded. Containing, also, an Alphabet and an Introduction, illustrating the Utility of the System and its Method of Use. By F. L. Crane, D. D. S. of Easton, Pa.

As will be seen by the above, Dr. Crane of Easton, Pa., has presented the profession with a case-book. It is beautifully gotten up, and most admirably arranged for the record of cases. It at the same time answers the purpose of a day-book and leger. By the adoption of a set of indicators, the necessity of writing out the operation in full is wholly avoided, and any one of these indicators may be made with a single touch of the pen. We like the arrangement of the book much, and take great pleasure in recommending it to the profession.

The publication of so many case-books argues well for the profession; it shows that its members feel the importance of collecting facts and making observations which may be applied to purposes of practical utility. We are glad that such a spirit of inquiry is abroad, as it must contribute to the advancement of the science and art of this most important branch of physical alleviation. It also shows that the ranks of the profession are beginning to be filled with scientific men—men who would elevate it to the dignity of the other branches of medicine.

The case-book of Dr. Crane contains about four hundred pages, and is bound in morocco. For the very beautiful copy which he has presented to us, we tender him our thanks.—*Balt. Ed.*

